# Genomic characterization and phylogenetic placement of Matryoshka RNA virus 1 associated with Plasmodium vivax malaria in Africa

**DOI:** 10.1099/acmi.0.001183.v4

**Published:** 2026-09-15

**Authors:** Frank Chilombolwa Nyondo

**Affiliations:** 1 Department of Biomedical Sciences, Ridgeway Campus, University of Zambia, School of Health Sciences, Lusaka, Zambia

**Keywords:** Matryoshka RNA virus, metatranscriptomics, malaria, MaRNAV, *Plasmodium vivax*, RNA-dependent-RNA polymerase

## Abstract

*Plasmodium vivax is* a major cause of human malaria. It harbours Matryoshka RNA virus 1 (MaRNAV-1), a bi-segmented positive-sense RNA virus. MaRNAV-1 was first described in *P. vivax* and is now recognized as part of a wider group of Matryoshka viruses. These viruses also infect other haemosporidian parasites such as *Leucocytozoon* and *Haemoproteus*. The presence of MaRNAV-1 in African-origin human *P. vivax*, however, has not been clearly established. This study investigated whether MaRNAV-1 is present in public African-origin *P. vivax* transcriptomic datasets. Any viral sequences recovered were characterized using comparative genomic and phylogenetic analyses. A secondary *in silico* analysis targeted African-origin *P. vivax* RNA-seq runs from public repositories. Although the search covered Africa, only Ethiopian datasets could be confidently identified, retrieved and compiled at the time. After quality control and screening for MaRNAV-1 RNA-dependent RNA polymerase (RdRp) signals, three high-confidence runs were selected for further analysis. Reference-guided reconstruction, ORF prediction, blast-based validation and RdRp phylogenetic analysis were performed. MaRNAV-1 was identified in three Ethiopian *P. vivax* malaria transcriptomes. This was supported by strong segment-level mapping, near-complete coverage, high mean depth and minimal low-depth masking. The recovered genomes showed the expected bisegmented organization of MaRNAV-1. Segment I was highly conserved and encoded the canonical RdRp in all three consensus sequences. Segment II showed the conserved organization of two overlapping hypothetical ORFs in all three consensus sequences. Blast analyses confirmed close similarity to MaRNAV-1 reference sequences. Phylogenetic inference grouped the Ethiopian sequences within the broader *P. vivax*-associated MaRNAV-1 lineage, alongside other recognized MaRNAV lineages distinct from more divergent narna-like viruses. These findings provide genomic evidence for MaRNAV-1 in publicly available African-origin *P. vivax* transcriptomic datasets and add to the emerging evidence for the virus in the African malaria context.

**Impact Statement**

This study shows that a lesser-known virus, Matryoshka RNA virus 1 (MaRNAV-1), can be detected in African *Plasmodium vivax* samples. *P. vivax* is one of the parasites that causes malaria in humans. MaRNAV-1 has not previously been clearly documented in publicly available African-origin *P. vivax* transcriptomic datasets. Its recovery from Ethiopian datasets therefore extends the documented occurrence of MaRNAV-1 to African-derived *P. vivax* RNA-seq data. It highlights the need for broader sampling to define its geographic distribution. The study shows malaria parasites may carry their own viruses, which could affect how they live, spread or interact with the human body. Although the work does not prove MaRNAV-1’s role, it provides a strong starting point for future studies. It also demonstrates that publicly available sequencing data can help answer new biological questions without requiring immediate new samples. The findings contribute to research showing that parasites often operate within complex biological systems that include their own viruses. African *P. vivax* remains understudied, so identifying MaRNAV-1 in malaria-related data will encourage future work to determine this virus’s prevalence, its occurrence in other malaria parasites and its impact on malaria biology and disease.

## Data Summary

 Metatranscriptomic RNA-sequencing datasets analysed in this study are publicly available and originate from *Plasmodium vivax* patient samples previously described by Kepple *et al*. [1]. Run accessions and associated metadata for the 3 RNA-seq datasets and 19 other Matryoshka and Narnaviral genomes used for comparative genomics and phylogenetic analysis are provided in Table S1 (available in the online supplementary material). Consensus Matryoshka RNA virus 1 segment sequences reconstructed from the retained RNA-seq runs have been deposited in the European Nucleotide Archive under BioProject PRJEB107815 with accession ranges OZ409293–OZ409371 and OZ409368–OZ409370, accessible at: https://www.ebi.ac.uk/ena/data/view/OZ409293 – RdRp (segment I): Pv01 https://www.ebi.ac.uk/ena/data/view/OZ409366 – RdRp (segment I): Pv02

https://www.ebi.ac.uk/ena/data/view/OZ409370 – RdRp (segment I): Pv03 https://www.ebi.ac.uk/ena/data/view/OZ409371 – Hypothetical Protein (segment II): Pv01 https://www.ebi.ac.uk/ena/data/view/OZ409368 – Hypothetical Protein (segment II): Pv02 http://www.ebi.ac.uk/ena/data/view/OZ492749 – Hypothetical Protein (segment II): Pv03

Derived analysis outputs supporting this study are additionally available on Figshare: coverage-informed consensus nt FASTA sequences (https://doi.org/10.6084/m9.figshare.31081816), predicted ORF FASTA sequences (https://doi.org/10.6084/m9.figshare.31081831) and phylogenetic tree generation code (https://doi.org/10.6084/m9.figshare.31081846).

## Introduction

Viruses in microbial eukaryotes are now seen as key components of parasite systems [2]. In several protozoan models, parasite-associated viruses affect parasite fitness, host immune responses and disease expression. These findings suggest that such viruses can influence parasite biology beyond what is revealed by parasite-focused studies [2–5]. The best-studied case, the Leishmania RNA virus, is associated with more severe inflammatory pathology and worse clinical outcomes. This shows the broader biological importance of parasite virology [6, 7].

Within this broader context, the identification of Matryoshka RNA virus 1 (MaRNAV-1) in human *Plasmodium vivax* expanded the scope of parasite-associated virology to include haemosporidian parasites [8]. MaRNAV-1 is a bisegmented positive-sense RNA virus, with segment I encoding the RNA-dependent RNA polymerase (RdRp) and segment II encoding non-polymerase hypothetical proteins, typically represented as overlapping ORFs [8]. This genome architecture is evolutionarily significant because it links MaRNAV-1 to narna-like viruses via its polymerase segment, while distinguishing it from classical narnaviruses, which are minimalist RNA replicons lacking a capsid and typically encoding only an RdRp [8, 9]. Since the initial description of MaRNAV-1, related Matryoshka viruses have been reported in other haemosporidian parasites, particularly *Leucocytozoon* and *Haemoproteus*. These findings suggest the existence of a broader, yet incompletely characterized, assemblage of parasite-associated narna-like viruses [10–12]. However, several important questions remain unresolved, including the geographical distribution of MaRNAV-1 in human *P. vivax*, the extent of conservation across its two genomic segments and the broader significance of its relationship to other narna-like viruses. More recently, the documented distribution of MaRNAV-1 has been extended by global mapping across Southeast Asia, South America and Oceania, again confined to *P. vivax* [13], while a preprint awaiting peer review has reported the first direct evidence that the virus infects the parasite and is associated with increased parasite transmission potential and host inflammatory responses [14]. The presence and diversity of MaRNAV-1 in African-origin human *P. vivax*, however, have remained largely unexamined at the transcriptomic level, which the present study addresses.

African *P. vivax* represents a pertinent context for investigating these questions. Although *P. vivax* has historically been underrecognized in sub-Saharan Africa due to the emphasis on Duffy-negativity and the predominant focus on *Plasmodium falciparum*, accumulating evidence now demonstrates that vivax malaria occurs in multiple African regions, including populations previously considered largely resistant to infection [15–19].

This study was conceived to broadly address African-origin *P. vivax* transcriptomic datasets, to determine whether MaRNAV-1 is present in publicly available African *P. vivax* data and to contextualize any recovered sequences within a comparative framework. However, at the time of analysis, the only *P. vivax* transcriptomic datasets from Africa that could be confidently identified, retrieved and screened were of Ethiopian origin. Also, Ethiopia is relevant in this context because *P. vivax* has long been recognized there, and recent genomic studies have shown that Ethiopian and broader East African parasite populations are epidemiologically and evolutionarily informative [20–22]. Ethiopia, therefore, emerged not only as the available African transcriptomic resource for this study but also as a biologically appropriate setting for investigating MaRNAV-1 in African *P. vivax*. In this way, the study provides additional sequence-level evidence for MaRNAV-1 in African-origin *P. vivax* transcriptomic data, supporting its presence in African-associated vivax malaria datasets and laying the groundwork for broader investigation of its distribution, diversity and biological relevance in African malaria-endemic settings.

## Methods

### Study design and datasets

This study was a secondary *in silico* analysis of publicly available transcriptomic sequencing data. The aim was to detect and characterize the genomic segments of MaRNAV-1. The *P. vivax* RNA-seq datasets analysed were generated in the primary transcriptomic study by Kepple *et al*. [1] and are available under NCBI SRA BioProject PRJNA784582. Because the present work focused on African-origin *P. vivax* transcriptomic data, inclusion was restricted to runs from African samples that could be confidently identified and retrieved from public repositories at the time of analysis. Using these criteria, 12 transcriptomic SRA runs made up the full set of African-origin datasets that could be located and successfully compiled for screening.

### Download and compilation of the public read dataset

Twelve African-origin *P. vivax* RNA-seq runs were retrieved from the European Nucleotide Archive (ENA) using its Portal Application Programming Interface. At the time of data collection, these were the only publicly available African-origin *P. vivax* transcriptomic datasets suitable for this analysis. For each accession, paired-end FASTQ locations, MD5 checksums and file sizes were programmatically obtained. Paired FASTQ files (R1/R2) were downloaded from ENA-hosted endpoints and organized by run-specific directories.

### Read preprocessing

Raw paired-end reads from 11 of the 12 retrieved runs were preprocessed using fastp v1.0.1 [23], with the software’s default trimming and filtering configuration. One run, SRR17069093, was excluded prior to quality control (QC) due to mismatched paired-end read files. In brief, bases with Phred scores below 15 were considered unqualified. Reads were excluded if more than 40% of bases were unqualified, if they contained more than 5 ambiguous bases (*N*) or if their length after trimming was below 15 bp. No average-quality threshold was applied. Low-complexity filtering was not enabled. Per-run FastP quality-control outputs were inspected. This confirmed improved base-quality profiles, successful adapter removal and acceptable read retention before downstream mapping and screening. The resulting trimmed paired FASTQ files were then used for MaRNAV detection screening and subsequent segment-resolved analyses.

### Screening for MaRNAV RdRp signal and selection of SRRs for complete analysis

To prioritize datasets with strong evidence of MaRNAV-1 RNA1 (RdRp), all fastp-processed SRRs were screened by aligning reads to the MaRNAV RdRp reference sequence [8]. Two primary metrics were summarized: (i) breadth of coverage across the full RdRp contig and (ii) mapped read support, including mean depth. SRRs with >90% RdRp breadth and >10× mean mapped depth were eligible for downstream analysis [24]. Priority was given to those with the strongest combined support profiles. Based on these criteria, the final SRRs retained for full segment-resolved variant calling and annotation were SRR17069092, SRR17069099 and SRR17069100.

### Reference-guided genome reconstruction

Reference-guided genome reconstruction was used to recover MaRNAV segment sequences from the cleaned and retained SRR datasets. This approach enabled reliable recovery of corresponding viral segments across samples, consistent generation of consensus sequences and direct comparison of sequence features and coding regions using uniform reference coordinates [25]. Established MaRNAV-1 segment references were selected, as MaRNAV-1 is currently the only named MaRNAV reported in association with human *P. vivax*, making it the most appropriate reference for targeted reconstruction [8, 13, 25]. Quality-controlled reads were aligned independently to MaRNAV-1 segment references, and mapped viral reads were used to generate sample-level consensus segment sequences.

### Reference sequences and rationale for four-segment reference FASTAs

MaRNAV-1 is a bisegmented positive-sense RNA virus with two functionally distinct genome segments. Segment I encodes the RdRp, essential for viral replication. Segment II encodes non-polymerase proteins, including a hypothetical protein/ORF2-like product, often represented as overlapping ORFs [8, 12]. Two closely related MaRNAV reference variants are available in GenBank. Four reference accessions were used, corresponding to two segments for each of the two reference variants:

- MN698828.1 and MN698829.1: RdRp segment references
- MN698826.1 and MN698827.1: non-RdRp hypothetical protein/ORF2-like segment references

Each SRR was processed independently for each segment reference to reduce ambiguity from cross-segment mapping and to enable consistent cross-run comparisons.

### Segment-resolved read alignment and Binary Alignment Map (BAM) processing

For each retained SRR, quality-filtered paired-end reads were independently aligned to each of the four MaRNAV reference accessions using Bowtie2 v2.5.4 [26] in very-sensitive local mode. Alignments were saved as BAM files, then sorted and indexed with samtools v1.23 [27]. Indexing facilitated rapid retrieval of reads from any region of the reference and was necessary for coverage and depth summarization as well as consensus reconstruction. Reference length (e.g. MN698828.1=2,934 nt) and per-contig mapped read counts were verified using samtools idxstats.

### Coverage profiling and low-depth masking

Per-base depth was calculated for each SRR segment BAM to identify regions of each reference segment supported by sufficient read coverage. Consensus generation was restricted to SRR–segment mappings with greater than 90% coverage breadth across the target reference segment [28, 29]. Positions with read depth below 10 were masked to ‘N’ to prevent weakly supported bases from being interpreted as confident nt calls [27]. Low-depth intervals were recorded as Biological Entity Dictionary (BED) masks, providing an auditable record of masked regions. This masking approach also prevents over-interpretation at the protein level, as codons spanning masked ‘N’ positions translate to ambiguous aas (‘X’), indicating low nt support rather than true aa substitutions [30].

### Variant calling and standardized ‘recall’ VCF generation

Variants were called independently for each SRR segment using a pileup-based workflow implemented in bcftools (version 1.23, using htslib 1.23), assuming a haploid model (ploidy=1) appropriate for viral genomes [27]. Variants were then recalled from the final BAMs and written as compressed, indexed VCFs. Variant burdens (SNP and indel counts) were summarized per SRR×segment. These procedures quantified differences between each run and the chosen reference segment, generated consistent variant representations across runs and segments for downstream processing and provided standardized variant inputs necessary for reproducible consensus sequence reconstruction and interpretation.

### Consensus reconstruction

Masked consensus sequences were generated for each SRR×segment by applying recalled variants to the corresponding reference using bcftools consensus, with the depth<10 BED mask replacing low-confidence positions with ‘N’ [31]. Consensus FASTA files were then used for downstream ORF prediction and comparative analyses, providing run-specific sequences with sufficient coverage and enabling comparison across runs and segments. Consensus lengths were checked per segment, and indel-associated length shifts were recorded to flag changes that could affect ORF boundaries.

### ORF prediction and protein translation

ORFs were predicted from consensus FASTA files using EMBOSS getorf v6.6.0.0 [32]. For RdRp segment consensuses, ORFs were extracted using the -find 1 option with a minimum size threshold appropriate for long coding regions (e.g. -minsize 450). For hypothetical protein segment II consensus sequences, parameters were adjusted to retain shorter, potentially overlapping coding regions.

### blast-based sequence validation

To validate the identity of SRR-derived MaRNAV consensus segments and predicted protein products, similarity searches were conducted on the NCBI web platform using NCBI blast. Blastn was used to compare nt sequences to the NCBI Core nt database; blastx compared translated nt consensus sequences in all six frames to the NCBI ClusteredNR protein database; and blastp compared translated ORF aa sequences to the NCBI ClusteredNR. All searches were performed using default blast parameters. For each query, results were assessed by percent identity, query coverage (or aligned length) and E-value. Top hits confirmed concordance with MaRNAV-related references and supported interpretation of the RdRp and hypothetical protein segments. Full blast outputs were saved as text files, and key results were summarized in tables for transparency.

### Assembly of the RdRp protein dataset and phylogenetic analysis

A comparative RdRp protein dataset was assembled to contextualize the Ethiopian MaRNAV-1 sequences within the broader diversity of MaRNAV and related narna-like viruses. The three Ethiopian RdRp aa sequences (OZ409293 – Pv01; OZ409366 – Pv02; OZ409370 – Pv03) generated in this study were used as successive queries against the NCBI non-redundant protein database using protein blast (blastp). For each query, the top 100 protein hits were screened, and sequences were retained if they exhibited at least 90% query coverage, at least 30% aa identity and a strong E-value indicative of significant similarity [33]. The corresponding protein accessions were retrieved in FASTA format, merged and de-duplicated to produce the reference protein set for downstream phylogenetic analysis.

### Phylogenetic inference and tree visualization

RdRp protein sequences were aligned using MAFFT v7.526 [34] with the --localpair and --maxiterate 1000 options. All-gap alignment columns were removed before phylogenetic inference. The primary alignment included 22 taxa, comprising the 3 Ethiopian MaRNAV-1 sequences and 19 representative reference RdRp proteins.

Maximum-likelihood phylogenetic analysis was performed in IQ-TREE v3.0.1 [35] with automatic aa model selection (ModelFinder). Branch support was evaluated using 1000 SH-aLRT and 1000 ultrafast bootstrap replicates with -bnni [36, 37]. Branch lengths represent aa substitutions per site. The resulting tree was midpoint-rooted for display.

The resulting maximum-likelihood tree was midpoint-rooted for visualization in R [8, 38]. Tree plotting and annotation were performed using R phylogenetics packages [39], with internal node labels showing SH-aLRT/UFBoot support values and tip labels displaying virus names, accession identifiers and reported host/source context.

## Results

### Dataset retrieval, QC and screening identified three *P. vivax* runs for complete MaRNAV-1 analysis

**Fig. 1.**
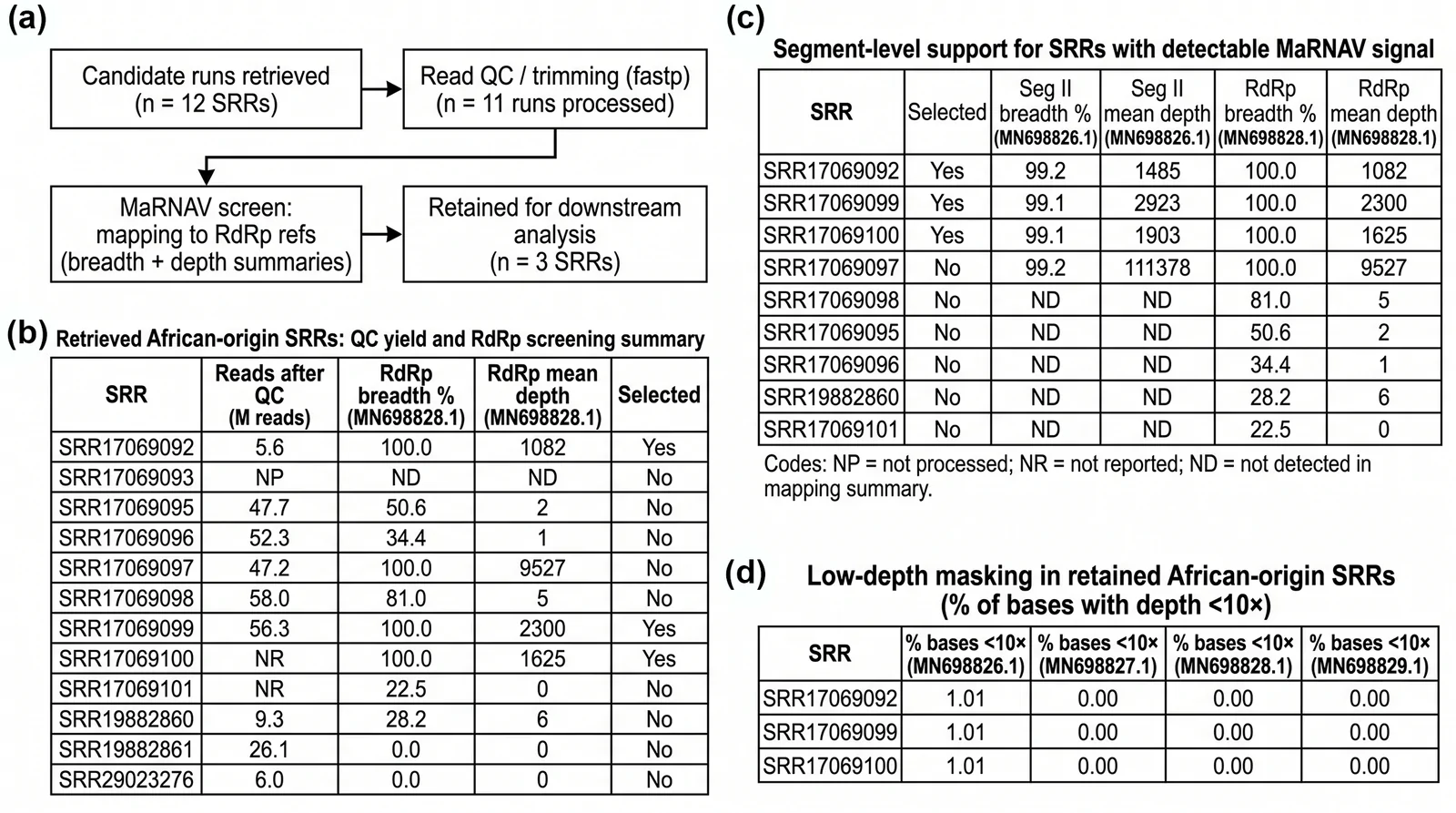
Selection of *P. vivax* RNA-seq runs and mapping support for Ethiopian MaRNAV consensus/ORF inference. (a) Workflow summarizing dataset retrieval (*n*=12 SRRs); read QC and trimming with fastp (*n*=11 runs processed); screening for MaRNAV signal by mapping reads to MaRNAV reference segments; and retaining a final set for downstream analyses, including consensus sequence generation, ORF prediction and phylogenetic analyses (*n*=3 SRRs). (b) For each SRR, QC yield shows the number of reads retained after fastp filtering (in millions). Screening metrics include breadth (the percentage of reference positions with ≥1 read) and mean depth (the average per-base read depth across the RdRp segment of reference MN698828.1). (c) Segment-level support for SRRs with detectable MaRNAV signal, showing breadth and mean depth for the hypothetical proteins (RNA2) segment reference (MN698826.1) and the RdRp segment reference (MN698828.1), using the same definitions as in panel b. (d) Low-depth masking summary for retained SRRs across the four MaRNAV reference accessions used in this study (RNA2: MN698826.1 and MN698827.1; RdRp: MN698828.1 and MN698829.1), reported as the percentage of reference positions with depth <10×. This threshold was used during consensus generation to mask low-support positions (to ‘N’), reducing the risk of treating weakly covered bases as confident nts and improving the interpretability of downstream translated ORFs.

Searches of the public archives identified 12 *P. vivax*-related metatranscriptomic RNA-seq runs of African origin, representing the full set yielded by the search strategy used in this study. These candidate runs were retrieved and screened for MaRNAV-1 signal. Read QC/trimming outputs were available for 11 of these runs (Fig. 1a). At the same time, SRR17069093 was not processed and therefore lacked downstream QC and screening metrics (Fig. 1b). Mapping-based screening against the MaRNAV-1 RdRp reference (MN698828.1) showed heterogeneous support across runs (Fig. 1b).

Three runs – SRR17069092, SRR17069099 and SRR17069100 – were retained for downstream consensus generation, ORF inference and phylogenetic analyses (Fig. 1a and b) based on strong RdRp breadth and depth profiles together with consistent segment-level support (Fig. 1c). RdRp breadth was 100% for SRR17069092 (mean depth 1,082×), SRR17069099 (2,300×) and SRR17069100 (1,625×) (Fig. 1b and c).

SRR17069097 also demonstrated complete RdRp breadth (100%), an exceptionally high mean depth (9,527×), and 47.2 million reads after QC (Fig. 1b), along with strong segment-level support (Fig. 1c). However, the depth and coverage profile for this run substantially exceeded those of the retained runs, resulting in its classification as a high-leverage outlier. To maintain comparability within the retained cohort and minimize outlier-driven bias in downstream analyses and interpretations, SRR17069097 was excluded from further analysis. Consequently, all subsequent analyses focused exclusively on the three retained African-origin runs, in accordance with the study workflow (Fig. 1a).

### Segment-level mapping support and low-depth masking summaries across MaRNAV references

Across the retained runs, segment-level mapping support was consistent for both viral segments: RdRp (segment I) and hypothetical protein/s (segment II) references (Fig. 1c). For segment II (MN698826.1), breadth and mean depth were 99.2%/1,485× (SRR17069092), 99.1%/2,923× (SRR17069099) and 99.1%/1,903× (SRR17069100) (Fig. 1c).

Low-depth masking summaries (depth <10×) showed minimal low-depth fractions across the three core runs, with 1.01% of sites <10× on MN698826.1 and 0.00% on MN698827.1, MN698828.1 and MN698829.1 across SRR17069092, SRR17069099 and SRR17069100, respectively (Fig. 1d).

### Genome organization and ORF architecture of Ethiopian MaRNAV consensuses

**Fig. 2.**
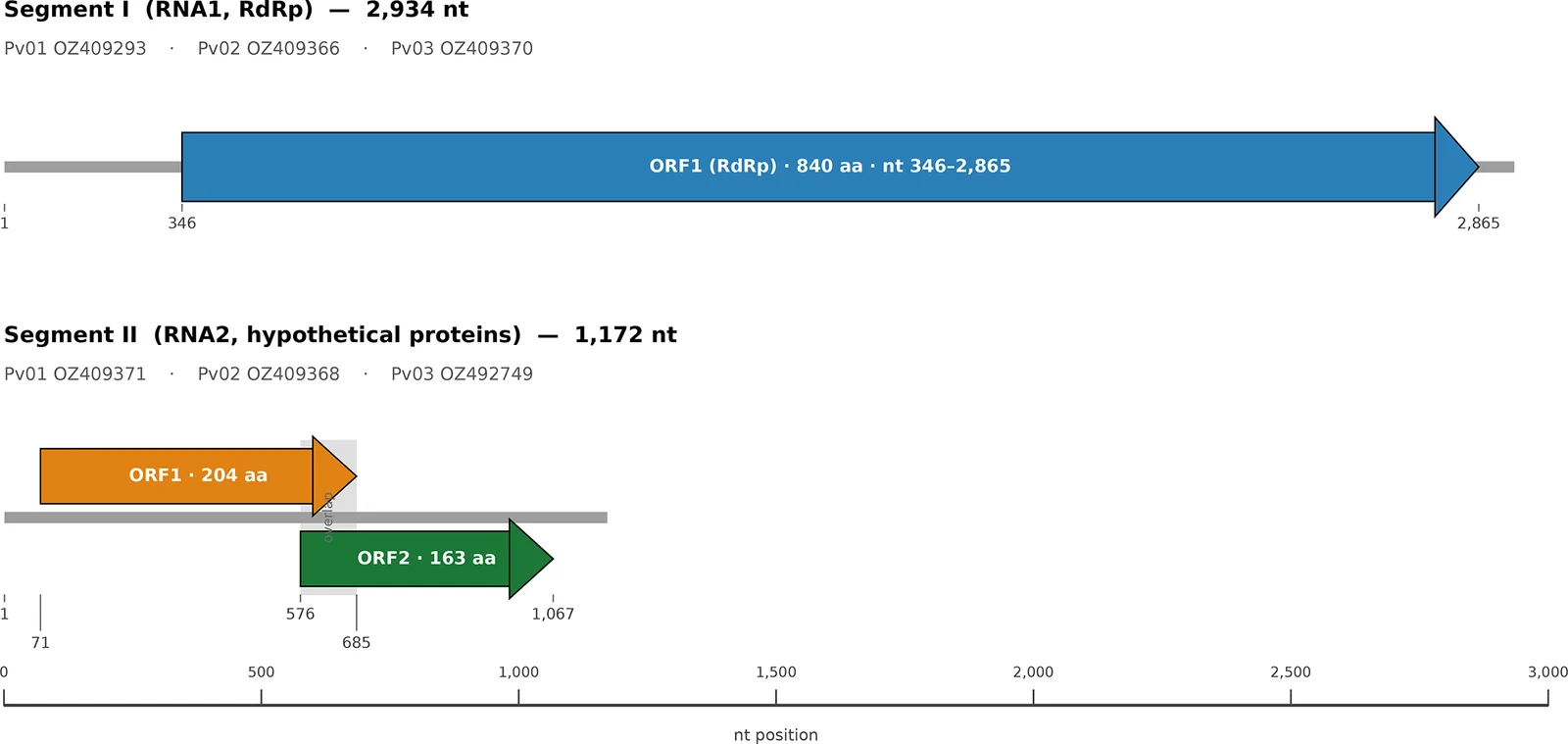
Genome organization of MaRNAV-1 sequences recovered from Ethiopian *P. vivax* transcriptomic datasets. Schematic representation of the two genomic segments of MaRNAV-1 reconstructed from three Ethiopian *P. vivax*-associated RNA-seq datasets. Segment I (RNA1; 2934 nt) contains a single large ORF encoding the RdRp, spanning nt 346–2,865 and corresponding to 840 aa in Pv01 (OZ409293), Pv02 (OZ409366) and Pv03 (OZ409370). Segment II (RNA2; 1,172 nt) contains two predicted overlapping ORFs encoding hypothetical proteins: ORF1 spans nt 71–685 and encodes a 204-aa product, while ORF2 spans nt 576–1,067 and encodes a 163-aa product. The two RNA2 ORFs overlap by 109 nt, from nt 576 to nt 685. Arrows indicate coding orientation and are drawn relative to nt position; colours distinguish the RdRp ORF on RNA1 from the two predicted hypothetical-protein ORFs on RNA2.

ORF annotation of the Ethiopian MaRNAV-1 consensus sequences (Pv01 – OZ409293; Pv02 – OZ409366; Pv03 – OZ409370) showed a conserved bisegmented genome organization consistent with MaRNAV-1 (Fig. 2; File S2). In all three consensuses, segment I comprised a single long ORF corresponding to the canonical RdRp coding region, spanning nt 346–2,865 and encoding an 840 aa protein on reading frame +1 (Fig. 2). This conserved architecture indicates strong structural preservation of the MaRNAV-1 RNA1 segment across the Ethiopian sequences.

Segment II, which encodes the non-RdRp putative hypothetical protein component of the MaRNAV-1 genome, showed a conserved overlapping-ORF organization across the three consensuses. In Pv01 – OZ409371 and Pv02 – OZ409368, two partially overlapping ORFs were identified on the positive strand, with ORF1 spanning nt 71–685 (204 aa) and ORF2 spanning nt 576–1,067 (163 aa), generating an overlap across nt 576–685 (Fig. 2). This organization was consistent between the two consensuses and supports a shared segment II coding architecture.

In Pv03 (OZ492749), the same two overlapping ORFs were recovered after correction of the consensus sequence. The originally deposited Pv03 consensus carried a single-nt insertion near the start of ORF1 that shifted the reading frame and introduced a premature stop codon, truncating the predicted product. Re-calling the consensus from the underlying reads (SRR17069100) with indel normalization recovered a full-length ORF1. It did not reproduce the inserted base, indicating that it had arisen during the original reference-guided reconstruction. After correction, ORF1 and ORF2 of Pv03 matched the coordinates and overlap observed in Pv01 and Pv02, indicating a shared segment II coding architecture across all three Ethiopian consensuses.

### blast-based validation confirms MaRNAV-1 identity of African consensus sequences

NCBI blastp analyses of translated ORFs supported assignment of the Ethiopian consensuses to MaRNAV-1. RdRp ORFs (840 aa) from Pv01–Pv03 showed 100% query coverage, 96.73–98.15% aa identity and *E*-values of 0.0 against MaRNAV-1 reference polymerases (File S3). Segment II hypothetical proteins likewise returned 100% query coverage with 96.02–98.04% aa identity and highly significant *E*-values (≤2×10^−^¹¹⁵), with top hits consistently annotated as MaRNAV-associated hypothetical proteins (File S3). Detailed NCBI blast reports were compiled into one folder and deposited in Figshare under DOI: 10.6084/m9.figshare.31082044.

### Global RdRp phylogeny situates African *P. vivax*-associated MaRNAV-1 within broader MaRNAV diversity

**Fig. 3.**
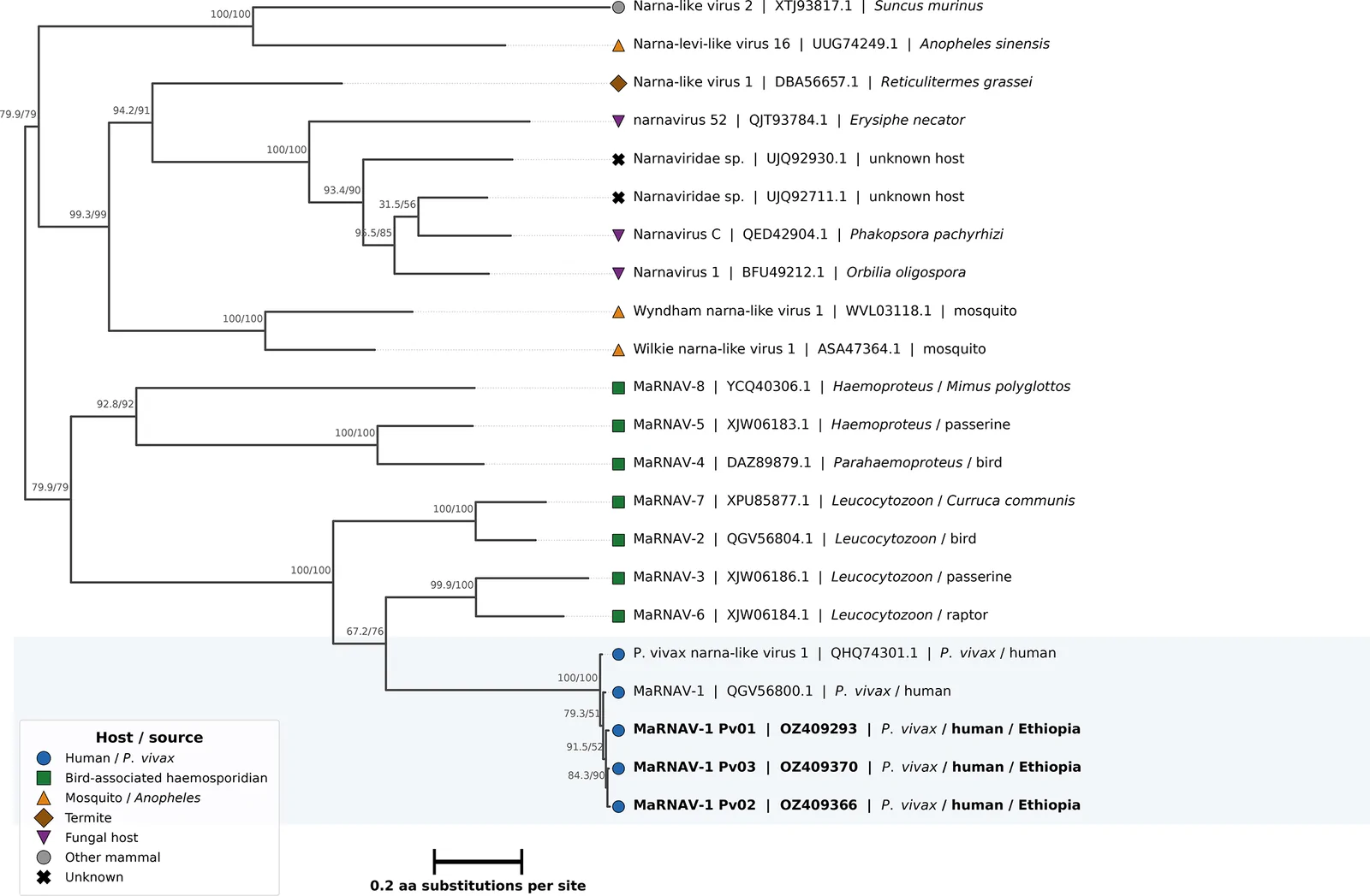
Midpoint-rooted maximum-likelihood phylogeny of MaRNAV and related narna-like viruses based on RdRp aa sequences. The tree was inferred from an alignment of 22 RdRp protein sequences, comprising the 3 Ethiopian *P. vivax*-associated MaRNAV-1 sequences generated in this study and 19 reference MaRNAV or narna-like virus sequences. Maximum-likelihood inference was performed using IQ-TREE with automatic aa model selection. The tree was midpoint-rooted for visualization. Internal node labels show SH-aLRT/UFBoot support values, and branch lengths represent aa substitutions per site. Tip labels show virus name, accession identifier and reported host/source context. Coloured symbols indicate host/source categories: human/*P. vivax*, bird-associated haemosporidian parasites, mosquito/*Anopheles*, termite, fungal host, other mammal or unknown. The shaded region highlights the *P. vivax*–associated cluster containing the Ethiopian MaRNAV-1 RdRp sequences recovered in this study, the previously reported *P. vivax* MaRNAV-1 reference and *P. vivax* narna-like virus 1. The three study-derived Ethiopian MaRNAV-1 sequences are shown in bold.

Maximum-likelihood phylogenetic analysis of RdRp aa sequences placed the three Ethiopian sequences (OZ409293, OZ409366 and OZ409370) within the MaRNAV 1 lineage (Fig. 3). The three study-derived sequences formed a compact cluster together with the *P. vivax*–associated reference QGV56800.1, supporting their placement within the broader human-associated *P. vivax* MaRNAV-1 clade. Although the precise internal branching order within this small cluster was not uniformly strongly supported, its overall placement within the MaRNAV 1 region of the tree was stable and clearly distinct from the other MaRNAV lineages.

Outside the Ethiopian *P. vivax* cluster, the tree resolved additional MaRNAV lineages associated with other haemosporidian groups. A *Haemoproteus*-associated avian cluster comprised MaRNAV 8 (YCQ40306.1), MaRNAV 5 (XJW06183.1) and MaRNAV 4 (DAZ89879.1). At the same time, two separate *Leucocytozoon*-associated avian groupings were also evident, namely MaRNAV 2 (QGV56804.1) with MaRNAV 7 (XPU85877.1) and MaRNAV 6 (XJW06184.1) with MaRNAV 3 (XJW06186.1) (Fig. 3). These patterns indicate that, within the MaRNAV assemblage, RdRp sequence relationships broadly track the reported haemosporidian associations.

More divergent narna-like and Narnaviridae-related sequences formed a separate part of the tree, distinct from the MaRNAV lineages. Within this broader assemblage, the sequences from *Anopheles sinensis* and from the shrew *Suncus murinus* formed a sister pair. In contrast, the termite-associated sequence (narna-like virus 1) occupied a nearby but separate position among other non-MaRNAV narna-like viruses (Fig. 3). Several additional sequences in this region were associated with fungal hosts, further emphasizing the broader evolutionary separation between the haemosporidian-associated MaRNAV clade and other narna-like viruses. Branch lengths are shown as aa substitutions per site, and internal node labels are reported as SH-aLRT/UFBoot support values.

## Discussion

This study demonstrates that MaRNAV-1 is detectable in African-origin *P. vivax* transcriptomes. Specifically, we identified three high-confidence MaRNAV-positive Ethiopian *P. vivax* RNA-seq runs. Full-genome reconstruction of these runs revealed that their sequences align closely with the known MaRNAV-1 genomic and phylogenetic frameworks. These genomes showed consistent segment-level mapping, near-complete coverage, high depth, minimal low-depth masking, strong blast concordance and stable phylogenetic clustering within the *P. vivax*-associated MaRNAV-1 lineage. Collectively, these results add to the emerging evidence for MaRNAV-1 in African *P. vivax* [13, 14].

The significance of the African context lies in the historically underrecognized status of *P. vivax* in sub-Saharan Africa. This is primarily due to the focus on Duffy-negative blood groups and the predominance of *P. falciparum* in malaria research [15, 40]. However, accumulating molecular and epidemiological evidence now demonstrates that *P. vivax* is present across multiple African regions, is even associated with complicated malaria cases and has been reported in populations previously considered resistant to infection [16–18, 41–43]. Ethiopia, in particular, has emerged as a key setting for understanding *P. vivax*. Both historically and in recent genomic studies, the parasite is clearly established and evolutionarily relevant [20–22]. In this context, the detection of MaRNAV-1 in Ethiopian *P. vivax* transcriptomes suggests that the biology of African *P. vivax* may encompass a virological component that remains largely unexplored.

A principal strength of this study is the use of a deliberately conservative analytical framework for detection and reconstruction. Public transcriptomic datasets are increasingly valuable for RNA virus discovery. The RdRp is the most reliable marker for assessing RNA virus diversity [44, 45]. Nevertheless, secondary mining of RNA-seq data is susceptible to uneven coverage, computational artefacts, ambiguous host assignment and over-interpretation of weak signals [30, 31, 46]. In this study, these risks were mitigated through explicit breadth-and-depth screening, low-depth masking during consensus generation, blast-based validation and phylogenetic confirmation. This conservative approach, to a certain extent, justifies the in-depth analysis of only three runs. The study prioritized high-confidence, internally comparable MaRNAV-positive datasets. After screening for MaRNAV-1 RdRp signal, only the transcriptomic datasets SRR17069092, SRR17069099 and SRR17069100 exhibited complete RdRp breadth, strong mapped depth and consistent support across viral segments. These factors enabled robust consensus generation and ORF interpretation. Although SRR17069097 also demonstrated strong viral support, its markedly deeper depth profile classified it as a high-leverage outlier, and it was excluded to minimize distortion in downstream comparisons [47].

The choice of reference-guided reconstruction for MaRNAV discovery from retained *P. vivax* transcriptomes was based on the fact that MaRNAV-1 is currently the only named Matryoshka virus reported from human *P. vivax* [8, 13]. This makes the established MaRNAV-1 segment references the most appropriate framework for sample-level consensus reconstruction and cross-sample comparison [8]. Under these conditions, reference-guided reconstruction offered clear practical advantages. It enabled consistent recovery of corresponding genomic regions across samples, standardized variant calling against uniform coordinates and direct comparison of coding features between the Ethiopian consensuses [27].

The recovered genomes reveal key findings regarding MaRNAV-1. Segment I is highly conserved across all three Ethiopian consensuses, encoding the canonical 840-aa RdRp at the expected genomic position and in the expected reading frame. This conservation underscores the essential functional role of viral polymerases and indicates that RdRp genes are generally more constrained than other genomic regions in preserving core catalytic and structural functions [48, 49]. Thus, segment I serves as the conserved replication backbone of MaRNAV-1. Segment II was likewise conserved across all three Ethiopian consensuses, with Pv01 (OZ409371), Pv02 (OZ409368) and Pv03 (OZ492749) sharing the same arrangement of two overlapping hypothetical ORFs. An apparent reduction initially observed in Pv03 was traced to a single-nt insertion introduced during reference-guided consensus reconstruction, which truncated ORF1 *in silico* but was not reproduced when the consensus was re-called from the underlying reads. After correction, Pv03 conformed to the architecture of the other two consensuses [8]. More generally, ORF prediction in compact RNA genomes is sensitive to local variation, masking and consensus uncertainty, as illustrated by the Pv03 insertion, underscoring the importance of read-level verification when interpreting apparent architectural differences [30].

This segment-level contrast is clearer when MaRNAV-1 is compared to narnaviruses and other narna-like viruses. Classical narnaviruses are typically regarded as minimalist RNA replicons, often lacking a capsid and generally encoding only an RdRp [9]. In this context, MaRNAV-1 retains a narnavirus-like evolutionary backbone in its conserved polymerase segment. It differs from canonical RdRp-only narnaviruses in possessing a second genomic segment encoding hypothetical non-polymerase proteins [8, 12]. The Ethiopian data support this interpretation: segment I functions as a conserved narna-like replication module, and segment II, although encoding proteins of unknown function, was likewise conserved across the three consensuses. This observation fits the hypothesis that Matryoshka viruses represent a more complex or modular narna-like form. In such viruses, a minimalist ancestral replication system may have acquired or retained an accessory genomic segment that could provide functions absent from classical narnaviruses [8, 9]. Although this study does not resolve the origins of this modularity, it supports an evolutionary model. In this model, the replication machinery remains conserved, while the function of the accessory genome segment remains to be established [8, 12].

The phylogenetic analysis further supports this interpretation. The RdRp dataset incorporated the three Ethiopian MaRNAV-1 sequences, additional recognized MaRNAV lineages and more divergent narna-like and Narnaviridae-related sequences. Within this framework, the Ethiopian sequences clustered within the MaRNAV-1 lineage alongside the previously described *P. vivax*-associated reference, while other MaRNAV lineages, such as MaRNAV 2 through MaRNAV 8, formed distinct clusters associated with other haemosporidian parasites [8, 10–12]. More divergent narna-like viruses occupied a separate region of the phylogenetic tree. This distinction demonstrates that the Ethiopian sequences are not merely generically narna-like but are part of a broader Matryoshka assemblage that is evolutionarily related to, yet clearly distinct from, other narna-like viruses. The resolution of multiple MaRNAV lineages in the tree further suggests that diversification within the Matryoshka group may broadly reflect haemosporidian association. Rather than being randomly distributed across the phylogeny, MaRNAVs linked to *Plasmodium*, *Leucocytozoon* and *Haemoproteus* tend to occupy related but distinct positions, implying that evolutionary divergence within the group may have proceeded in parallel with association to different haemosporidian parasite lineages [8, 10, 12].

The current data permit careful consideration of biological origin. While contamination cannot be entirely ruled out in secondary analyses of public RNA-seq data, the preponderance of evidence supports a *P. vivax*-associated origin of the recovered MaRNAV-1 signal – the retained Ethiopian datasets from the *P. vivax* transcriptomic study by Kepple *et al*. [1] included both MaRNAV-1 genome segments, which showed strong, concordant support in these runs. In addition, all the reconstructed ORFs yielded high-similarity MaRNAV-1 hits by blast, and the Ethiopian RdRp sequences clustered within the previously described *P. vivax*-associated MaRNAV-1 lineage [1, 8]. Nevertheless, this inference should equally be approached with caution. This study did not experimentally localize the virus within *P. vivax or* determine parasite burden in the analysed transcriptomes. Although Kepple *et al*. [1] reported an overall parasitaemia of ~4,000 parasites μl^−1^ for the Ethiopian source infections, sample-specific parasite-load values were not available for SRR17069092, SRR17069099 and SRR17069100. Therefore, it was not possible to assess whether MaRNAV-1 recovery correlated with parasite abundance in these samples. Recent work has begun to clarify the biology of this association. In a preprint that has not yet completed peer review, MaRNAV-1 was reported to be an intracellular virus infecting *P. vivax* across blood, sporozoite and liver stages, with higher viral loads associated with increased parasite transmission potential, reflected by greater gametocyte abundance and higher mosquito-stage oocyst burdens, and with stronger host inflammatory responses [14]. If corroborated through peer review, these findings would indicate that MaRNAV-1 is a genuine parasite-infecting virus rather than an incidental associate. The present *in silico* analysis of public transcriptomes can establish association but not infection, and the localization and functional consequences of MaRNAV-1 in these Ethiopian samples remain to be determined directly.

The strengths of this study must be weighed against its clear limitations. This study is a secondary *in silico* analysis of a limited number of public blood-stage transcriptomes and was not prospectively designed to establish viral localization, intracellular distribution, transmission route or functional impact. The use of reference-guided reconstruction, while appropriate for the analysis’s targeted aims, imposes important constraints on inference. Because reconstruction was anchored to known MaRNAV-1 segments, the pipeline was inherently more effective at recovering closely related MaRNAV-1-like genomes than at detecting highly divergent Matryoshka-like viruses, unexpected segment architectures, novel insertions or genomic organizations absent from the reference set [50, 51]. In this context, a *de novo* assembly strategy might have been more sensitive to unanticipated viral forms, whereas the present approach prioritized targeted recovery, consistency and cross-sample comparability [26, 27].

Another limitation is that phylogenetic inference was based only on the RdRp-encoding segment. Although RdRp is the most conserved and most readily comparable module across RNA viruses and was therefore well suited to placing the Ethiopian sequences within broader MaRNAV diversity [52, 53], it represents only the replication-associated component of a bisegmented genome. As a result, the phylogeny may not fully capture the evolutionary signal present in segment II, which was not included in the phylogenetic analysis. Any segment-specific divergence, reassortment-like history or lineage-specific change affecting the non-RdRp segment would therefore not be resolved by an RdRp-only tree [51]. Furthermore, the biological interpretation of segment II remains limited by the lack of experimentally validated protein functions [8, 12, 54].

Another important implication relates to the breadth of malaria species in Africa. Current evidence supports the presence of MaRNAV-1 in *P. vivax* and related Matryoshka viruses in other haemosporidian parasites. Still, Matryoshka viruses have not been documented in *P. falciparum* in the literature reviewed here [8, 10–12]. This gap represents a significant direction for future research, particularly in African regions where *P. falciparum* is predominant. Comprehensive screening of African *P. falciparum* transcriptomic and metatranscriptomic datasets, employing prospectively defined and fully reported analytical pipelines, will be essential to determine whether the apparent absence of Matryoshka viruses in *P. falciparum* reflects a true biological restriction, undersampling or methodological bias. Recent global mapping of MaRNAV-1 is consistent with such a restriction, detecting the virus across Southeast Asia, South America and, for the first time, Oceania, but only in metatranscriptomes containing *P. vivax* and not in screened *P. falciparum* samples, including those of African origin [13].

In summary, the Ethiopian genomes recovered in this study reinforce the understanding of MaRNAV-1 as a specialized narna-like virus with a conserved RdRp core and a second, less-characterized genomic segment that may encode its most distinctive biological features. The findings are consistent with this model: segment I was structurally stable and phylogenetically coherent, and segment II showed the same conserved overlapping-ORF architecture across all three consensuses, although the function of its encoded proteins remains uncharacterized. From an evolutionary perspective, this supports the hypothesis that MaRNAV-1 shares ancestry with narna-like viruses but has diverged through increased genomic complexity [8, 9]. Biologically, the study extends this framework to African *P. vivax* and contributes to the literature by advancing from detection to comparative genomic interpretation. Future research should prioritize targeted molecular confirmation in independent African *P. vivax* cohorts, experimental validation of segment II coding features, localization of the virus within parasite material and broader species-level screening, including formal investigation of African *P. falciparum* infections, to determine whether MaRNAV-1 is merely a consistent viral associate of *P. vivax* or a biologically significant component of its ecology and pathobiology.

## Supplementary material

10.1099/acmi.0.001183.v4 Table S1.

10.1099/acmi.0.001183.v4 Supplementary Material 1.

10.1099/acmi.0.001183.v4 Supplementary Material 2.

## Abbreviations

AI: artificial intelligence
BAM: Binary Alignment Map
BED: Biological Entity Dictionary
ENA: European Nucleotide Archive
MaRNAV-1: Matryoshka RNA virus 1
protein blast: blastp
QC: quality control
RdRp: RNA-dependent RNA polymerase
